# Immunological Adverse Events After Autologous Hematopoietic Stem Cell Transplantation in Systemic Sclerosis Patients

**DOI:** 10.3389/fimmu.2021.723349

**Published:** 2021-09-03

**Authors:** Patrick-Pascal Strunz, Matthias Froehlich, Michael Gernert, Eva Christina Schwaneck, Anna Fleischer, Ann-Christin Pecher, Hans-Peter Tony, Joerg Christoph Henes, Marc Schmalzing

**Affiliations:** ^1^Department of Internal Medicine II, University Hospital of Wuerzburg, Wuerzburg, Germany; ^2^Department of Internal Medicine II, University Hospital of Tuebingen, Tuebingen, Germany

**Keywords:** scleroderma, fever, autoimmune disease, Grave’s disease, modified Rodnan skin score (mRSS), risk factor analysis, engraftment syndrome, Sjögren’s syndrome

## Abstract

Autologous hematopoietic stem cell transplantation (aHSCT) represents an effective treatment for systemic sclerosis (SSc), but it also can cause immunological adverse events (iAEs). Therefore, we aimed to determine the frequency of iAEs [engraftment syndrome (ES) and secondary autoimmune disorder (sAD)] and to identify potential risk factors for their development in a retrospective analysis on 22 patients similarly transplanted due to SSc. While nine patients (41%) suffered from ESs, seven sADs occurred in six patients (27%). Patients who developed ES were older in our cohort (52.45 *vs.* 42.58 years, p = .0433, Cohen’s d = 0.86), and cardiac involvement by SSc was associated with development of ES (OR = 40.11, p = .0017). Patients with manifestation of sAD had a higher modified Rodnan skin score (mRSS) reduction after aHSCT (90.50% *vs.* 60.00%, p = .0064, r = .65). Thus, IAEs are common after aHSCT for SSc and can occur in different stages during and after aHSCT with characteristic clinical manifestations. Good cutaneous response after aHSCT might be considered as a risk factor for sAD, and higher age at aHSCT and cardiac involvement might be considered as risk factors for the development of ES.

## Introduction

Since the ASSIST, ASTIS, and SCOT trials, autologous hematopoietic stem cell transplantation (aHSCT) is an established therapeutic option in the treatment of life- or organ-threatening systemic sclerosis (SSc) (1–8). In severe progressive forms of SSc, the superiority of aHSCT to conventional cyclophosphamide treatment was proven by those randomized controlled trials (RCTs) with respect to response, quality of life, and overall survival (1–3, 7, 8). However, aHSCT also constitutes a major impact on immunological homeostasis: possible immunological adverse events (iAEs) can be differentiated according to type, extent, mechanism, and time of occurrence into engraftment syndromes (ESs) and secondary autoimmune diseases (sADs). During the engraftment period and the white blood cell (WBC) recovery, ES can endanger the patient (9–13). These reactions are likely caused by the regeneration of leukocytes with following tissue invasion and triggering of autoinflammatory reactions (9–13). To date, no consistent risk factors for ES have been identified (9). Thus, one of our aims was to identify risk factors for development of ES in transplanted SSc patients. To the best of our knowledge, this is the first study of ES and its risk factors in the context of aHSCT in SSc. The second type of iAEs are sADs manifesting several months after WBC recovery (4–6). The exact mechanism of the development of sADs after aHSCT in SSc has not been fully understood yet. However, it can be assumed that genetic predisposition for autoimmunity and intense lymphocyte-depleting effects of the conditioning regimen with disturbance of the peripheral and central immune tolerance are involved (4, 5). Besides some case reports, two major surveys have investigated the frequency of sADs related to aHSCT for primary autoimmune diseases (pADs) in general, but without focusing on SSc (4, 5). Thus, our objective was to assess the occurrence of sADs and risk factors for sAD in a homogenous cohort of patients receiving aHSCT due to SSc.

## Method

### Study Design

A retrospective cohort study of all SSc patients in follow-up after aHSCT at our institution was performed to examine manifestations of iAEs during or after aHSCT. The patients underwent aHSCT either at the University Hospital of Tuebingen or at the University Hospital of Wuerzburg. Due to the retrospective nature of the study, ethical approval is not needed by German legislation. Informed consent is not required due to anonymous data collection and storage.

### Study Population

Ten male and 12 female patients (n = 22) suffering from SSc (two patients had an overlap syndrome between SSc with mixed connective tissue disease and myositis) are currently in follow-up at our center after receiving an aHSCT treatment. Their transplantations were performed between December 2008 and December 2019. End of recruitment was January 2020. One further transplanted patient had died due to complications after a survived cardiac arrest during aHSCT (this patient was not considered in the analysis and is not included in these 22 patients). The median age at transplantation was 47.57 years (total range 23.10–62.78 years). Besides cutaneous manifestation, 59% (n = 13) had a cardiac and 82% (n = 18) a pulmonary involvement by SSc. Cardiac manifestations included only mild-to-moderate involvement: whereas 39% (n = 5) had only asymptomatic troponinemia without other explanation (some with biopsy-proven involvement), systolic dysfunction was present in 15% (n = 2) and diastolic dysfunction in 46% (n = 6). Myocardial fibrosis on MRI was seen in 31% (n = 4), and relevant arrhythmias were also present in 31% (n = 4). Two patients were suffering from pulmonary arterial hypertension (PAH) (**Table 1**). For stem cell mobilization, cyclophosphamide was used, followed by the administration of granulocyte colony-stimulating factor (G-CSF) starting at day 4 (for the individual dosage, see **Table 2**). Then, all patients (except for two patients who received a combination of thiotepa, cyclophosphamide, and anti-thymocyte globulin (ATG) as conditioning regimen) were treated similarly according to a protocol containing cyclophosphamide (from day −5 till day −2, for the individual dosage, see **Table 2**) and ATG (for the individual dosage, see **Table 2**; 1 mg/kg of methylprednisolone was given as concomitant medication during ATG administration; total infusion time of ATG was 8–12 h under monitoring of vital parameters) with individual dose adjustment due to individual organ function followed by CD34+-selected stem cell transplantation (on day 0). In two patients, CD34+ selection could not be performed due to low stem cell counts after leukapheresis (see **Table 2**). G-CSF was only used for stem cell mobilization and not during aHSCT (except for one patient).

**Table 1:** Included patients.

Patient no.	Sex	Age at aHSCT	iAE	mRSS before aHSCT	Relative mRSS reduction	Involvement			Antibodies before aHSCT
						Cardiac	Pulmonary	PAH	
1	M	40.55	sAD	34	91%	−	−	−	Scl-70
2	F	43.83	sAD	31	90%	+^b^	+	−	None
3	M	57.2	ES	22	100%	+^b^	+	−	Scl-70
4	F	27.77	sAD	21	91%	−	+	−	Scl-70, SSA
5	F	31.79	None	nk	nk	−	+	−	Scl-70
6	M	38.46	None	26	69%	−	+	−	Scl-70
7	F	46.96	None	14	57%	−	+	−	None
8	F	44.35	ES	26	85%	+^b^	+	−	Scl-70
9	F	23.1	None	28	68%	+^b^	−	−	Scl-70, RF
10	F	38.81	ES	9	78%	+	+	−	Scl-70, SSA
11	F	53.04	sAD	28	68%	+	−	−	SSA, U1-RNP, RNP70
12	M	58.24	ES	41	66%	+	+	−	Scl-70
13	F	29.64	sAD	15	87%	−	+	−	Scl-70
14	M	53.82	sAD	nk	nk	−	+	−	Scl-70, PM/SCL, RF
15	F	56.92	None	20	45%	−	+	+	None
16	M	48.18	ES	32	56%	+	+	−	RP11/155
17	M	58.03	ES	44	61%	+	+	−	Scl-70
18	F	43.83	ES	5	60%	+	+	−	Scl-70
19	M	60.66	ES	12	58%	+	+	−	Scl-70
20	M	55.22	None	40	40%	+	−	+	Scl-70
21	F	52.41	None	12	42%	−	+	−	Scl-70, RF
22	M	62.78	ES	16	31%	+	+	−	Scl-70

List of all included patients sorted by their age at aHSCT, their sex, the type of observed immunological adverse event, their mRSS before aHSCT, their relative mRSS reduction in percent, their organ involvement (pulmonary, cardiac, and PAH), and their autoantibody pattern before aHSCT.

M, male; F, female; ES, engraftment syndrome; sAD, secondary autoimmune disorder; aHSCT, autologous hematopoietic stem cell transplantation; nk, not known; iAE, immunological adverse event; mRSS, modified Rodnan skin score; PAH, pulmonary arterial hypertension; RF, rheumatoid factor.

b Biopsy proven.

**Table 2:** Individual transplantation data.

Patient no.	CD34 × 10^6^/kg bw	Mobilization treatment	Conditioning regimen	Date of aHSCT
1	4.39	2 × CYC 2 g/m^2^	4 × CYC 50 mg/kg + 3 × ATG 2.5 mg/kg	August 2009
2	3.86	2 × CYC 1.5 g/m^2^	2 × CYC 50 mg/kg + 2 THT 5 mg/kg, 3 × ATG 10 mg/kg	March 2011
3	3.75	2× CYC 2 g/m^2^	2 × CYC 50 mg/kg + 2 THT 5 mg/kg, 3 × ATG 10 mg/kg	December 2008
4	3.12	2× CYC 2 g/m^2^	4 × CYC 50 mg/kg + 3 × ATG 10 mg/kg	October 2009
5	nk	CYC	CYC + ATG	June 2009
6	3.00	2 × CYC 1 g/m^2^	4 × CYC 50 mg/kg + 4 × ATG 5/10/10/10 mg/kg*	September 2013
7	5.20	2 × CYC 1 g/m^2^	4 × CYC 50 mg/kg + 4 × ATG 1/9/10/10 mg/kg*	July 2015
8	nk	2 × CYC 1.5 g/m^2^	4 × CYC 50 mg/kg + 4 × ATG 10 mg/kg	November 2010
9	2.55^u^	2 × CYC 1 g/m^2^	4 × CYC 50 mg/kg + 4 × ATG 1/9/10/10 mg/kg*	March 2015
10	3.50	2 × CYC 1 g/m^2^	4 × CYC 50 mg/kg + 4 × ATG 1/9/10/10 mg/kg*	June 2015
11	3.88	2 × CYC 1 g/m^2^	4 × CYC 50 mg/kg + 4 × ATG 1/9/10/10 mg/kg*	February 2016
12	2.93^u^	2 × CYC 1 g/m^2^	4 × CYC 50 mg/kg + 4 × ATG 1/9/10/10 mg/kg*	August 2016
13	3.22	CYC	4 × CYC 50 mg/kg + 3 × ATG 2.5 mg/kg	September 2009
14	2.60	2 × CYC 1.5 g/m^2^	4 × CYC 50 mg/kg + 4 × ATG 10 mg/kg	October 2010
15	7.02	2 × CYC 1.5 g/m^2^	4 × CYC 50 mg/kg + 4 × ATG 1/9/10/10 mg/kg*	June 2017
16	3.16	2 × CYC 1 g/m^2^	4 × CYC 50 mg/kg + 4 × ATG 1/9/10/10 mg/kg*	February 2018
17	2.59	2 × CYC 1 g/m^2^	4 × CYC 50 mg/kg + 4 × ATG 1/9/10/10 mg/kg*	March 2018
18	4.40	2 × CYC 1 g/m^2^	4 × CYC 50 mg/kg + 4 × ATG 1/9/10/10 mg/kg*	January 2018
19	2.50	2 × CYC 1 g/m^2^	4 × CYC 50 mg/kg + 4 × ATG 1/9/10/10 mg/kg*	February 2018
20	3.17	2 × CYC 1 g/m^2^	4 × CYC 40 mg/kg + 4 × ATG 1/4/5/5 mg/kg*	March 2018
21	2.90	2 × CYC 1 g/m^2^	4 × CYC 50 mg/kg + 4 × ATG 1/4/5/5 mg/kg*	October 2019
22	4.00	2 × CYC 1 g/m^2^	4 × CYC 50 mg/kg + 4 × ATG 1/4/5/5 mg/kg*	December 2019

bw, body weight; aHSCT, autologous hematopoietic stem cell transplantation; CYC, cyclophosphamide; THT, thiotepa; ATG, anti-thymocyte globulin; nk, not known.

*Dose per day; i.e., 4 × ATG 1/9/10/10 mg/kg means 1 mg/kg at day 1, 9 at day 2, and 10 at days 3 and 4.

^u^CD34+ unselected.

### Definitions and Diagnosis

We divided the observed phenomena into ES and sADs.

For ES, various catalogues of diagnostic criteria exist (9–13). Due to their focus on clinical symptoms and their higher sensitivity (13), we decided to use the Maiolino criteria [“non-infectious fever plus any of the following: skin rash, pulmonary infiltrates or diarrhea” (12)] as basis for the diagnosis of ES (9, 11, 12): non‐infectious fever (>38°C) accompanied by at least one further ES-typical symptom (rash, pulmonary infiltrates, and encephalopathy) occurring during WBC recovery (defined as the first of three consecutive days with absolute neutrophil count (ANC) >0.5 ×10^9^/L after the first post-transplant nadir). Two cases of non-infectious fever and musculoskeletal symptoms during regeneration were also counted as ES or ES-like due to their close temporal relationship with engraftment and after exclusion of other causes (see *Discussion*).

The diagnosis of sADs was made by experienced rheumatologists in the field of stem cell transplantation on the basis of classic diagnostic criteria and clinical evaluation. Disease-specific autoantibodies were first detectable after aHSCT.

### Variables, Data Collection, and Data Processing

Patients’ medical history is stored at our clinical documentation software and was adopted from it for analysis. Based on pathophysiological considerations, we chose cell dose of CD34+ cells/kg body weight, age at aHSCT, sex, pulmonary or cardiac involvement, time to WBC recovery (defined as first of three consecutive days with absolute leukocyte count >0.5 ×10^9^/L after the first post-transplant nadir) in days after aHSCT, and summit/maximum of leukocytes level after aHSCT in n/µl as independent variables for risk factor analysis of ES. Only complete datasets were included in the evaluation: while complete information to age and sex was available, time to WBC recovery, maximum of WBC increase, and CD34+ cell dose, only 19, 15, and 20 datasets could be used for analysis. For assessment of risk factors of sAD, we used the modified Rodnan skin score (mRSS) before aHSCT, the relative reduction of mRSS by transplantation (mRSS before aHSCT − best mRSS after aHSCT/mRSS before aHSCT) in percent, age at aHSCT, positivity for Scl-70, and sex as risk factors for sAD. While for age, sex, and positivity of Scl-70 complete datasets were available, for mRSS before aHSCT and relative reduction of mRSS, only 20 datasets were complete and could be considered in analysis. One female patient developed two independent autoimmune diseases, so that this was considered as two separate cases in the analysis.

### Statistical Analysis

Continuous values were tested for normal distribution by performing the Shapiro–Wilk test and the Kolmogorov–Smirnov test (only when n was too low for analysis by the Shapiro–Wilk test). When values were normally distributed, means and standard deviations (SDs) were calculated, and differences were analyzed with a two-sided unpaired t-test. When normal distribution could not be determined, medians with interquartile ranges (IQRs) were calculated, and the Mann–Whitney test was performed to detect differences between groups. For the categorical scaled variables, contingency table analysis, the odds ratios, confidence intervals, and significances were calculated. For testing of significance in odds ratios, Fisher’s exact test was used. These calculations were performed using Prism (Version 5.01, August 7, 2007). Differences were considered significant when p-values were less than 0.05, and p <.1 was described as a trend toward significance. When significant differences could be observed, a statistical effect size was then computed to check the statistical effect of the observed difference: for the Mann–Whitney test, we used Pearson’s correlation coefficient (r = |z/√N|); and for unpaired t-test, we performed Cohen’s d (d = ((x_1_) − (x_2_))/√σ) (14, 15). Cohen’s d > 0.8 and Pearson’s r >.5 were considered as a strong effect size (14, 15). For calculation of effect sizes, SPSS from IBM (Version 26.0.0.0) was used.

## Results

### Engraftment Syndrome in Context With aHSCT for SSc

Nine of 22 transplanted patients (41%) showed signs of ES (see **Table 3**): all of them presented with fever (>38°C) during WBC recovery without causal relationship to infections or SSc disease activity. Pulmonary symptoms as respiratory failure and idiopathic pneumonia syndrome were seen in three patients (33%). A rash occurred in two patients (22%), and one patient developed an encephalopathy with agitation, confusion, and aphasia (11%). Two patients (22%) developed musculoskeletal symptoms with arthritis and myalgia. While spontaneous recovery without further drug intervention occurred in one patient, eight out of nine patients (89%) received causal therapy: all these patients were treated with systemic glucocorticoids (GCs) or GC-based topical skin therapy. The patient with arthritis received anakinra additionally to taper prednisolone and to stop further autoinflammation. All patients finally recovered from ES without sequelae.

**Table 3:** Patients with onset of engraftment syndrome (ES).

Patient no.	Age	Sex	Symptoms	Pre-CRP^2^	ES-CRP^2^	Treatment
3	57.2	M	Febrile syndrome during WBC recovery^1^	nk	nk	No specific treatment
8	44.35	F	Idiopathic pneumonia^1^	nk	nk	GCs (1 mg/kg)
12	58.24	M	Non-infectious fever (38.6°C), carpal arthritis beginning during engraftment	5.86	8.83	GC (prednisolone 15 mg), HCQ, anakinra, colchicine
16	48.18	M	Non-infectious fever (39.8°C), sudden respiratory failure (SpO_2_ = 79%), and pulmonary infiltration	0.78	24.32	GC (100 mg prednisolone)
17	58.03	M	Non-infectious fever (38.6°C), edema, rash (erythroderma)	2.96	6.20	Topical and systemic GCs (prednisolone 20 mg)
18	43.83	F	Non-infectious fever (38.6°C), myalgia	0.42	3.80	No specific treatment
19	60.66	M	Non-infectious fever (39.4°C), encephalopathy with aphasia	0.10	4.15	GC (dexamethasone 20 mg)
22	62.78	M	Non-infectious fever (38.4°C), diarrhea, sudden respiratory failure (SpO_2_ = 84%), and pulmonary infiltration	0.38	12.90	GC (prednisolone 20 mg)
10	38.81	F	Non-infectious fever (38.2°C), maculopapular rash	0.21	0.55	Topical GC

List of all patients with engraftment syndrome sorted by sex, age at transplantation, symptoms, pre-aHSCT CRP (at admission for aHSCT), CRP at manifestation of ES, and received treatment for ES. All patients had fever and increased CRP (relative to baseline CRP at admission) during manifestation of ES.

S, sex; ES, engraftment syndrome; GC, glucocorticoid; HCQ, hydroxychloroquine; SpO_2_, oxygen saturation measured by pulse oximetry; nk, not known; aHSCT, autologous hematopoietic stem cell transplant; CRP, C-reactive protein.

^1^Adopted diagnoses from patients’ medical reports.

^2^In mg/dl, reference range < 0.5 mg/dl.

**Figure 1:**
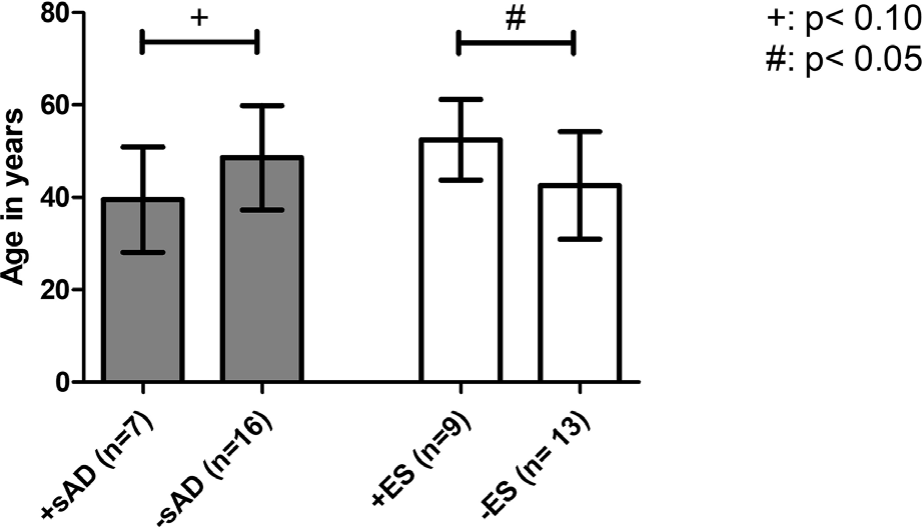
Age at aHSCT as a risk factor for developing an iAE. Comparison of the mean age at aHSCT as a risk factor between transplanted SSc patients with the onset of ES (+ES) and without the onset of ES (−ES) and patients with the onset of sAD (+sAD) and without sAD (−sAD). Older mean age at aHSCT was found to be associated with the development of ES. iAE, immunological adverse event; aHSCT, autologous hematopoietic stem cell transplantation; ES, engraftment syndrome; sAD, secondary autoimmune disease; SSc, systemic sclerosis. Error bars are given as SD.

Another aim was to define potential risk factors for the development of ES after aHSCT: the mean age at aHSCT was significantly higher in the group with ES (52.45 ± 8.70 years *vs.* 42.58 ± 11.63 years, p = .0433) and had a strong statistical effect size (Cohen’s d = 0.86) (**Figure 1**). The other investigated parameters CD34+ cell dose, maximum of leukocytes’ increase after aHSCT, and duration from aHSCT until WBC recovery in days showed no significant differences between the case and control groups. While cardiac involvement by SSc was associated with development of ES (OR = 40.11, 95% CI 1.885 to 853.6, p = .0017), patients with pulmonary involvement had no significant higher risk of ES in our odds ratio analysis. Male sex was not associated with a higher risk for ES (OR = 4.5, 95% CI 0.7297 to 27.75, p = .1920) (see **Table 4**).

**Table 4:** Risk factor analysis for ES.

Risk factor	ES	No ES	p
Mean age at aHSCT (years)	52.45 ± 8.70	42.58 ± 11.63	.0433
Median of CD34+ cell dose (×10^6^ cells/kg)	3.330 (IQR, 1.263)	3.195 (IQR, 1.338)	.6713
Mean time to WBC recovery (days)	12.43 ± 1.40	12.83 ± 2.37	.6872
Mean of max. leukocyte increase (n/µl)	14,600 ± 5,543	10,640 ± 4,441	.1482
**Risk factor**	**Odds ratio**	**95% confidence interval**	**p**
Male sex	4.5	0.7297 to 27.75	.1920
Pulmonary manifestation	9.00	0.4229 to 191.5	.1150
Cardiac manifestation	40.11	1.885 to 853.6	.0017

Risk factor analysis for engraftment syndrome. For metric scaled variables, means ± standard deviation or medians with interquartile range (IQR) are shown. Ordinal scaled variables are presented as odds ratio with 95% confidence interval. A higher mean age was found in the group with onset of engraftment syndrome. Cardiac involvement before HSCT was associated with the risk of developing ES. The other investigated variables showed no significant differences between the two groups.

ES, engraftment syndrome; WBC, white blood cell; HSCT, hematopoietic stem cell transplant.

### Secondary Autoimmune Diseases

During the follow-up, 27% of the patients (n = 6) developed a total number of seven sADs with a median latency between onset and aHSCT of 1.50 years (total range, 0.17–5.08 years): two patients (29% of all observed sADs) fell ill with Sjögren’s syndrome (SjS), two with Grave’s disease (29%), and one each with microscopic polyangiitis (MPA), Hashimoto’s thyroiditis, and immune thrombocytopenic purpura (ITP) (14% each) (**Table 5**). One female patient developed two independent autoimmune disorders (Grave’s disease and ITP). Interestingly, another female patient presented a new low titer (19 IU/ml) of anti-citrullinated protein antibodies (ACPAs) after aHSCT without having symptoms of active rheumatoid arthritis yet.

**Table 5:** Patients with onset of a secondary autoimmune disorder (sAD).

Patient no.	Sex	Age at HSCT	sAD	Antibody pattern	
				New after aHSCT	Before aHSCT
1	M	40.55	MPA with glomerulonephritis	MPO	Scl-70
2	F	43.83	Grave’s disease	TRAK, TPO, TGO	None
11	F	53.04	Hashimoto’s thyroiditis with rhabdomyolysis	TGO, TPO	SSA, U1-RNP, RNP70
13	F	29.64	Sjögren’s syndrome with sicca syndrome, bilateral parotitis	SSA	Scl-70
14	M	53.82	Sjögren’s syndrome with sicca syndrome, hypergammaglobulinemia with livedo reticularis	SSA, SSB	Scl-70, PM/SCL, RF
4	F	27.77	ITP		Scl-70, SSA
			Grave’s disease	TRAK	

List of all patients with secondary autoimmune disorders sorted by sex, age at transplantation, mRSS before HSCT, new detected autoantibodies after aHSCT, and antibody pattern before aHSCT.

M, male; F, female; mRSS, modified Rodnan skin score; MPA, microscopic polyangiitis; ITP, immune thrombocytopenic purpura; MPO, myeloperoxidase; TRAK, TSH receptor antibody; TPO, thyroid peroxidase antibody; TGO, thyroglobulin antibody; SSA, SSA(Ro) antibodies; SSB, SSB(LA) antibodies; RF, rheumatoid factor; aHSCT, autologous hematopoietic stem cell transplant.

**Figure 2:**
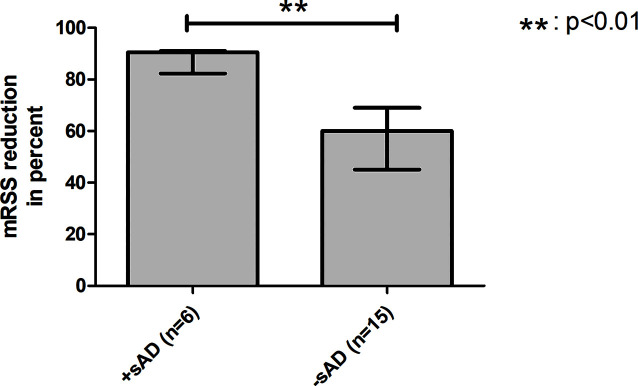
Better cutaneous response to aHSCT is a potential risk factor for sAD. Comparison between transplanted SSc patients with development of sAD and without development of sAD. The group with onset of sAD had a higher reduction of mRSS by transplantation, indicating that better cutaneous response to aHSCT might be a risk factor for developing sAD. aHSCT, autologous hematopoietic stem cell transplantation; mRSS, modified Rodnan skin score; sAD, secondary autoimmune disease; SSc, systemic sclerosis. Error bars are given as interquartile range (IQR).

Additionally, patients were examined to find potential risk factors for developing sAD: the mean age of the group developing sAD was numerically lower than in the group without sAD (39.49 ± 11.41 years *vs.* 48.56 ± 11.27 years, p = .0913) and showed a statistical trend toward significance. The severity of skin involvement expressed by the mRSS before aHSCT was not significantly different between the two groups (mean mRSS before aHSCT in the case group: 25.00 ± 7.18 *vs.* control group 23.13 ± 12.19, p = .7314). To determine whether the cutaneous response to aHSCT constitutes a risk factor for sAD, we also compared the patients’ relative reduction in mRSS as a surrogate parameter for therapy response: patients developing sAD showed a higher median reduction in mRSS than the control group (case group: 90.50% (IQR, 8.75%) *vs.* control group: 60.00% (IQR, 24.00%), p = .0064), and a strong effect size was found for that difference (r = .65) (**Figure 2**). Female sex (odds ratio 2.5, 95% CI 0.3699 to 16.90, p = .4050) and negativity for Scl-70 (odds ratio 1,733, 95% CI 0.2197 to 13.68, p = .6214) were not associated with a higher risk of sAD (**Table 6**).

**Table 6:** Risk factor analysis for sAD.

Risk factor	Odds ratio	95% confidence interval	p
Female sex	2.5	0.3699–16.90	.4050
Negativity for Scl-70	1.733	0.2197–13.68	.6214
**Risk factor**	**sAD**	**No sAD**	**p**
Mean of mRSS before aHSCT	25.00 ± 7.18	23.13 ± 12.19	.7314
Median of relative reduction of mRSS	90.50%	60.00%	.0064
	(IQR, 8.75%)	(IQR, 24.00%)	
Mean age at aHSCT (years)	39.49 ± 11.41	48.56 ± 11.27	.0913

Risk factor analysis for secondary autoimmune disorders. For metric scaled variables, means ± standard deviation or medians with interquartile range (IQR) are shown. Ordinal scaled variables are presented as odds ratio with 95% confidence interval. A higher median mRSS reduction by aHSCT was found in the group with onset of sAD. The other investigated variables showed no significant differences between the two groups.

sAD, secondary autoimmune disorders; mRSS, modified Rodnan skin score; aHSCT, autologous hematopoietic stem cell transplant.

## Discussion

This study represents the first SSc-specific analysis of manifestations of iAEs during or after aHSCT. The term engraftment syndrome was first defined by Lee et al. after a retrospective analysis of patients undergoing aHSCT for various diseases (9, 16): in their analysis, 59% developed immunological phenomena like fever, skin rash, capillary leak, and pulmonary infiltration occurring with a median onset of 7 days after aHSCT (9, 16). Two similar diagnostic criteria catalogues were suggested independently by Spritzer and Maiolino: these included non-infectious fever, skin rash, pulmonary infiltrates, diarrhea, encephalopathy, renal insufficiency, or hepatic failure with an onset during the engraftment period (9, 12, 17). As biomarkers, elevated C-reactive protein (CRP) levels are associated with ES (9, 13). ES has already been described in the context of aHSCT for pAD (multiple sclerosis) (13, 18). Therefore, we think that the data from our investigation fit into this context: 41% of our investigated SSc patients developed an ES with the typical manifestations as non-infectious fever with elevated CRP levels, skin rash, pneumonitis with respiratory failure, and encephalopathy. Interestingly, we observed two cases of patients with musculoskeletal symptoms accompanied by non-infectious fever manifesting during WBC recovery. These symptoms are not considered as a classical manifestation of ES compared with the criteria by Maiolino or Spritzer (9–12, 17). However, we claim that these might also be forms of ES specific for aHSCT in rheumatic inflammatory diseases due to the close temporal relationship with engraftment and after exclusion of other causes (11). Spontaneous resolution is described, but when medicamentous intervention becomes necessary, systemic GCs are recommended (9). Most of our patients could be treated sufficiently by the administration of prednisolone except for one case of a patient developing arthritis as the manifestation of his ES. When tapering GCs, his arthritis recurred so that the interleukin-1 receptor antagonist anakinra was used successfully. So far, defining risk factors for ES provided inconsistent results (9–11, 13). G-CSF treatment is discussed as a potential risk factor for ES development (9, 11), but only one of our patients received G-CSF after aHSCT. In our cohort, the mean age at aHSCT was significantly higher in the group with ES than in the control group, and patients with cardiac involvement had a much higher risk of developing ES than those without. Since the exact mechanisms leading to the development of ES are not well understood (9, 13), the explanation of the observed risk factors in our collective remains largely speculative: endothelial damage, release of pro-inflammatory cytokines, and invasion of neutrophils with degranulation are suspected elements of the ES pathogenesis (9, 10, 13, 17). Therefore, a higher age and cardiac manifestation might be associated with more cardiovascular or endothelial alterations and a more severe form of SSc, which might support releasing pro-inflammatory cytokines from the endothelium, stimulating leukocytes to invade during WBC recovery and induce ES (9–11, 13). Because ES has mostly been studied in aHSCT for malignancies (9–13, 16, 17), for which good cardiac and pulmonary function pre-aHSCT is a necessary condition, cardiac manifestation might have not been considered as a risk factor yet and, therefore, might be specific for aHSCT in pAD patients. An explanation for the higher mean age in the ES group might be immunosenescence/inflammaging: recent research work revealed a basal, subclinical, and non-infectious, age-related inflammation triggered by higher activation and expression of the NLRP3 inflammasome with consecutive higher basal IL1 production during senescence (19–22). IL1 is one of the cytokines suspected to play a crucial role in the pathogenesis of ES so that age-related disposition to IL1 production might support ES development (9–11). Additionally, elderly people show higher counts of neutrophils and pro-inflammatory monocytes (23). Since ES occurs during recovery of cells of the innate immune system and due to their suspected role in ES pathogenesis (9–13, 17), age-related alterations of these cells might also contribute to ES. In conclusion, these age-related predispositions to autoinflammation might explain the older mean age in the group with ES.

In a retrospective single-center study of 155 patients with various autoimmune diseases, Loh et al. looked for the occurrence of sAD depending on the conditioning regimen (4). A second, larger multicenter study by Daikeler et al. used data from the EBMT Autoimmune Disease Working Party to investigate the frequency of sAD after aHSCT: 347 patients after aHSCT due to different pADs were screened for developing of sAD (5). In both studies, SSc patients were included but without focusing on an SSc-specific context, like we did. In our study, 27% of the transplanted patients developed a sAD. Interestingly, this is a much higher frequency compared with the prevalence of 9% described by Daikeler et al. or 4% described by Loh et al. (4, 5). Both investigated cohorts included patients with various pADs. The prevalence of sAD in the SSc subgroup in the study by Daikeler and colleagues was also only 10%. The difference between our frequency and that of the other two studies cannot be explained by a distinction in the follow-up periods (up to 11 years in our cohort *vs.* 10 years at Loh and colleagues/15 years at Daikeler and colleagues) (4, 5). One cause for this higher prevalence might be the fact that we performed conditioning regimen with ATG and CD34+-selected aHSCT in all patients (except for 2), while Daikeler et al. and Loh et al. included patients with various conditioning regimens as well as CD34+-selected and CD34+-unselected grafts (4, 5). Daikeler et al. found in their analysis that the use of ATG accompanied by CD34+ selection is a risk factor for developing a sAD (5). CD34+-selected grafts are considered to interfere more with the immune reconstitution process and imbalances more the immune system than unmanipulated grafts by removing not only autoreactive T cells but also regulatory T cells (24, 25). Another possible explanation for that difference could be that our dataset is more detailed than that of Daikeler et al. by the fact that follow-up, data collection, and analysis were performed at the same center with unlimited and unfiltered access to patients’ data, while Daikeler et al. used data from EBMT Registry provided by multiple centers. Therefore, sADs might be underrepresented in the study by Daikeler et al. The median latency of 1.50 years from transplantation to disease manifestation differed not much from that reported by Daikeler et al. (1.83 years). While Daikeler et al. and Loh et al. mainly reported autoimmune cytopenia, thyroiditis, Grave’s disease, rheumatoid arthritis, and sarcoidosis in their included patients, we discovered SjS and Grave’s disease as the most frequent sADs occurring after aHSCT in our patients. Surprisingly, a case of new-onset MPA was noticed being by itself an extremely rare disease [this case was already published as a case report before (6)]. To the best of our knowledge, SjS has not been reported yet to be a typical autoimmune disorder following aHSCT for a pAD (4, 5). As a secondary form, SjS can be associated with other autoimmune connective tissue diseases (26). Therefore, a sicca syndrome after aHSCT would not be considered an independent primary disease, especially when the typical SSA(Ro) and SSB(LA) antibodies were preexisting before aHSCT (26). In our SjS patients, new developing sicca syndrome was accompanied by other severe symptoms, like bilateral parotitis or hypergammaglobulinemia-induced vasculopathy with livedo reticularis; and, strikingly, SjS-typical autoantibodies became first positive after aHSCT. Since patients’ SSc additionally showed an excellent response to aHSCT, we assessed these two cases as newly occurring SjS independent of the underlying disease.

In risk factor analysis, our study revealed a higher reduction of mRSS by aHSCT in the sAD group, indicating that a better cutaneous response to treatment might be a risk factor for manifestation of sAD. Distinctions in the reconstitution time of the different lymphocyte subsets leading to imbalance of the regenerating immune system, especially B cell preceding T-cell regeneration, a loss of peripheral tolerance, a failure of thymic negative selection, and genetic disposition are the discussed mechanisms of sAD development (4, 5, 27–31). Better cutaneous response might be associated with a higher grade of clearance of the autoreactive lymphocytes of pAD, which might emerge new arising autoreactive lymphocytes to occupy their slots/niches and induce sADs. While Loh et al. only looked for the influence of the conditioning regimen as a risk factor for the development of sAD, Daikeler et al. also defined other risk factors for sAD: systemic lupus erythematosus (SLE), short interval between diagnosis and aHSCT, and the use of ATG in combination with CD34+-selection were significant risk factors in their analysis. In contrast, all of our patients received the same conditioning regimen (except for two cases who received additional thiotepa), and only patients with SSc were included. In the study of Daikeler and colleagues, younger age was also reported to have a trend to be a risk factor for sAD with just missing significance (p = .062) (5). Interestingly, we made the same observation that the mean age in the case group was younger than the mean age of the control group with also just missing significance (p = .0913). In the context of sAD, the mean age at aHSCT behaved reciprocally as compared with ES, where an older mean age was found in the group with the onset of ES. This finding is controversial due to the fact that higher age is associated with autoimmunity (23). Daikeler and colleagues could also find no plausible explanation therefore and could only refer to a single-center study that reported a high incidence of sADs in newborns undergoing umbilical cord blood transplantation (5, 32).

### Limitations

The limiting factors of this trial are its retrospective character and its small number of involved patients in follow-up at a single center. Therefore, data interpretation has to be performed with particular care due to the small number of included patients (n = 22). The findings of this study have to be further validated in larger cohorts with more included patients. Despite these limitations, it offers the benefits of a homogeneous cohort of SSc patients, which received the same treatment protocol so that the influences of different treatment modalities and different pADs can be neglected in comparison with those of the other similar studies.

## Conclusion

In conclusion, we demonstrated that in SSc, aHSCT is associated with the development of different types of iAEs. ES is also present in aHSCT for other diseases, while the development of a sAD seems to be a particular risk if transplantation is due to a pAD. Transplant physicians should consider the possibility of ES development when fever and rise of inflammatory biomarkers occur during WBC recovery, and infectious causes can be excluded, especially in older patients and cardiac involvement pre-aHSCT. During follow-up after aHSCT, centers should be aware of the possibility for sADs. Patients should be screened routinely for the development of sAD, especially when they are young and have an excellent regression of skin fibrosis. The occurrence of sADs after aHSCT might necessitate an immunosuppressive therapy after aHSCT. aHSCT represents a very effective treatment of severe forms of SSc, and we hope to further improve its safety with the data of this work.

## Data Availability Statement

The original contributions presented in the study are included in the article/supplementary material. Further inquiries can be directed to the corresponding author.

## Ethics Statement

Ethical review and approval was not required for the study on human participants in accordance with the local legislation and institutional requirements. Written informed consent for participation was not required for this study in accordance with the national legislation and the institutional requirements.

## Author Contributions

Study design and conception: P-PS and MS. Data acquisition: P-PS, MS, and JH. Analysis and interpretation: P-PS, MF, MG, ES, AF, A-CP, H-PT, JH, and MS. Drafting: P-PS and MS. Revision: P-PS, MF, MG, ES, AF, A-CP, H-PT, JH, and MS. Final approval: P-PS, MF, MG, ES, AF, A-CP, H-PT, JH, and MS. All authors contributed to the article and approved the submitted version.

## Conflict of Interest

P-PS received travel grants from Janssen-Cilag and AbbVie. MF reported travel grants from AbbVie, Novartis, Janssen, and Eli Lilly and compensation for board memberships from AbbVie. MG received travel grants from AbbVie, Chugai, Eli Lilly, Hexal, Janssen-Cilag, Novartis, and Pfizer. Janssen, Eli Lilly, Chugai, Roche, Takeda (Shire), AbbVie, and Novartis supported ES. H-PT received speaker’s fees, travel grants, research funding, or compensation for consultancies or board memberships from AbbVie, BMS, Chugai/Roche, Eli Lilly, Gilead, Janssen, Novartis, Sandoz/Hexal, Sanofi Aventis, and Takeda (Shire). JH reported speaker’s fees, travel grants, and research funding from AbbVie, BMS, Boehringer/Ingelheim, Celgene, Chugai/Roche, Eli Lilly, Janssen-Cilag, Novartis, SOBI, Pfizer, and UCB. MS received speaker’s fees, travel grants, research funding, or compensation for consultancies or board memberships from AbbVie, Actelion, AstraZeneca, BMS, Boehringer/Ingelheim, Celgene, Chugai/Roche, Eli Lilly, Genzyme, Gilead, Hexal/Sandoz, Janssen-Cilag, MSD, Novartis, Pfizer, Sanofi Pasteur, Takeda (Shire), and UCB (less than $10,000 each).

The remaining authors declare that the research was conducted in the absence of any commercial or financial relationships that could be construed as a potential conflict of interest.

## Publisher’s Note

All claims expressed in this article are solely those of the authors and do not necessarily represent those of their affiliated organizations, or those of the publisher, the editors and the reviewers. Any product that may be evaluated in this article, or claim that may be made by its manufacturer, is not guaranteed or endorsed by the publisher.
